# Mucosa associated invariant T and natural killer cells in active and budesonide treated collagenous colitis patients

**DOI:** 10.3389/fimmu.2022.981740

**Published:** 2022-12-15

**Authors:** Niki Daferera, Sofia Nyström, Henrik Hjortswang, Simone Ignatova, Maria C. Jenmalm, Magnus Ström, Andreas Münch

**Affiliations:** ^1^Department of Gastroenterology, Faculty of Health Sciences, Linköping University, Linköping, Sweden; ^2^Department of Biomedical and Clinical Sciences, Faculty of Health Sciences, Linköping University, Linköping, Sweden; ^3^Department of Clinical Immunology and Transfusion Medicine, Linköping University, Linköping, Sweden; ^4^Department of Pathology, Linköping University, Linköping, Sweden

**Keywords:** collagenous colitis, budesonide, microscopic colitis (MC), MAIT cell, natural killar cells

## Abstract

**Introduction:**

Collagenous colitis (CC) is an inflammatory bowel disease, which usually responds to budesonide treatment. Our aim was to study the immunological background of the disease.

**Methods:**

Analyses of peripheral and mucosal MAIT (mucosa associated invariant T cells) and NK (natural killer) cells were performed with flow cytometry. Numbers of mucosal cells were calculated using immunohistochemistry. We studied the same patients with active untreated CC (au-CC) and again while in remission on budesonide treatment. Budesonide refractory patients and healthy controls were also included. The memory marker CD45R0 and activation marker CD154 and CD69 were used to further study the cells. Finally B cells, CD4^+^ and CD8^+^ T cells were also analysed.

**Results:**

The percentages of circulating CD56^dim^CD16^+^ NK cells as well as MAIT cells (CD3^+^TCRVa7.2^+^CD161^+^) were decreased in au-CC compared to healthy controls. This difference was not seen in the mucosa; where we instead found increased numbers of mucosal CD4^+^ T cells and CD8^+^ T cells in au-CC. Mucosal immune cell numbers were not affected by budesonide treatment. In refractory CC we found increased mucosal numbers of MAIT cells, CD4^+^ and CD8^+^ T cells compared to au-CC.

**Discussion:**

Patients with active collagenous colitis have lower percentages of circulating MAIT and NK cells. However, there was no change of these cells in the colonic mucosa. Most mucosal cell populations were increased in budesonide refractory as compared to au-CC patients, particularly the number of MAIT cells. This may indicate that T cell targeting therapy could be an alternative in budesonide refractory CC.

## Introduction

Collagenous colitis (CC) is an inflammatory bowel disease characterized by chronic non-bloody watery diarrhoea. Together with lymphocytic colitis, CC constitutes microscopic colitis which has a pooled prevalence of 119 per 100.000 person years, and a median age at diagnosis of 65 years (1).

CC pathophysiology is currently considered multifactorial, with environmental, luminal, immune and genetic factors contributing to the pathophysiology of the disease. The hallmarks of CC histology are increased infiltration of lymphocytes in the lamina propria and in the epithelium of the colonic mucosa, as well as a thickened collagen subepithelial band (>10μm) (2). The CC lamina propria is believed to be mainly infiltrated by CD4^+^ T-helper cells (3) and the epithelium by CD8^+^ cytotoxic T cells (3–5). While one study found an increased number of plasma cells in the lamina propria of CC patients (4), no difference in plasma immunoglobulin levels were noticed between CC and healthy controls (6). The lack of macroscopic inflammation, despite the immune cell infiltration of the mucosa, has previously been attributed to mucosal FoxP3^+^ T-regulatory cells (5). However, we recently reported that the expanded population of mucosal FoxP3^+^ T cells in CC most likely lack immune regulatory capacity (7).

The mainstay of CC treatment is oral budesonide which induces remission in 80% of patients (8, 9), with less than 5% being budesonide-refractory (10). In addition to the good response to budesonide therapy, increased eosinophilic mucosal infiltration (11–13), increased number of mast cells (14) and increased expression of the antimicrobial enzyme lysozyme (15) in CC mucosa also support an inflammatory aetiology of the disease. Furthermore, immunogenetic studies have shown that variants in tight junction genes (16), and in genes associated with the interleukin (IL)-6-174 GG genotype (17), as well as the HLA haplotype 8.1, are associated with CC (18).

Natural killer (NK) cells have historically been of great interest in mucosal immunology, and more recently mucosa associated invariant T (MAIT) cells have been discovered (19). Mucosal NK cells are involved in tissue homeostasis as well as in the induction of inflammatory responses (20). In Crohn’s disease increased frequencies of lamina propria NK cells have been reported and NK cells have been suggested a role in the disease pathophysiology (21). MAIT cells, present in the blood and in the gastrointestinal mucosa (22), are known to contribute to fibrinogenesis in the human kidney and liver (23, 24). Crohn’s disease is also characterized by increased numbers of mucosal MAIT cells (25, 26), while MAIT cells have been reported to be decreased in peripheral blood of ulcerative colitis and Crohns disease patients when compared to healthy controls (27). Knowledge of the distribution of various immune cell subsets in CC is missing. To our knowledge, NK cells and MAIT cells have never been studied in CC.

Our aim was to increase the understanding of the CC pathophysiology by extensive immune phenotyping of mucosal and circulating lymphocyte subsets with multi-colour flow cytometry, including NK cells and MAIT cells, in this prospective study of CC. We used histological samples of mucosal tissue for quantification of CD3^+^ T cell numbers. The circulating and mucosal distributions of lymphocyte subsets were compared between patients with active untreated CC and after budesonide-induced remission as well as with healthy controls. To further explore possible cellular different immune phenotypes in CC, budesonide refractory patients were also included in the study. The memory marker CD45R0 (28) and the activation marker CD154 (29) was used to characterise the circulating and mucosal cells in CC as well as the CD69 marker for activated circulating cells (30) and tissue resident mucosal cells (31).

We found that the percentages of circulating MAIT and NK subsets were decreased in au-CC patients compared to HC. The most pronounced findings were higher numbers of MAIT cells, CD8^+^ T cells, CD4^+^ T cells, as well as B cells, in budesonide refractory CC compared to au-CC.

## Methods

### Study population

Twenty-three patients with active untreated disease (au-CC) as well as twenty healthy controls (HC) and 12 budesonide refractory patients (ref-CC) were included in the flow cytometry study. Two of the 23 patients were found to be budesonide refractory and thus excluded from the active untreated CC group and instead included in the ref-CC group. Furthermore, 19 of 21 CC patients accepted a second investigation while being in remission on 9 mg budesonide treatment, **Supplementary Presentation 1**. The Hjortswang criteria were used to classify if patients were in active disease or in remission (32). All HC were in their 6^th^ decade of life and were included from the national colonic cancer screening program. A history of autoimmune diseases, recent treatment with antibiotics, any immunomodulating therapy and the presence of any other gastrointestinal disease, including irritable bowel syndrome were exclusion criteria for the HC group. The clinical characteristics of the study population are presented in **Table 1**.

**Table 1:** Demographics and clinical characteristics of patients and controls.

	HC	au-CC	rb-CC	ref-CC
**Total number of subjects**	20	21	19	14
**Sex, no (%) females**	9 (45)	17 (80)	15 (79)	12 (86)
**Age, mean (range)**	61 (60–71)	57 (27–86)	59 (27–86)	52 (25-75)
**Disease duration (years), mean (range)**	—–	6 (1–24)	6 (1–24)	4 (1–8)
**Collagen band mean in** *μm* **(range)**	—–	32 (12–50)	23 (2–51)	30 (10–50)
**Number of stools/day, mean (range)**	1 (0–3)	7 (5–12)	1 (1–2)	9 (4–15)
**Number of watery stools/day, mean (range)**	0 (0)	7 (4–10)	0 (0)	8 (4–15)
Autoimmune diseases no (%)				
Celiac disease	0 (0)	3 (14)	2 (10)	2 (14)
Thyroid disease	0 (0)	4 (19)	3 (15)	0 (0)
Diabetes type 2	0 (0)	1 (4)	1 (5)	0 (0)
Psoriasis	0 (0)	1 (4)	1 (5)	0 (0)
Polymyalgia rheumatic	0 (0)	1 (4)	1 (5)	0 (0)
Collagenous gastritis	0 (0)	1 (4)	1 (5)	0 (0)
Previous medication, no (%)				
Bile acid binders	0 (0)	6 (30)	6 (30)	9 (64)
Loperamide	0 (0)	16 (85)	16 (85)	11 (79)
Concomitant medication, no (%)				
Corticosteroids (Budesonide)	0 (0)	0 (0)	19 (100)	6 (43)

HC, healthy controls; CC, collagenous colitis; au, active untreated; rb, remission budesonide; ref, refractory.

### Sample collection

Sigmoidoscopies were performed in the morning hours at the endoscopy department of the Linkoping University hospital. Ten colonic biopsies were collected from the descending colon at an advanced sigmoidoscopy and the biopsies were immediately processed into single cell suspensions. Six biopsies were also obtained for histological analysis. All study participants received the same laxative (Picoprep, Ferring). Peripheral whole blood samples were collected in EDTA (ethylenediaminetetraacetid acid) tubes and processed in parallel.

### Mucosal intraepithelial and lamina propria lymphocyte isolation

The colonic biopsies were incubated with Hanks balanced salt solution (ThermoFisher scientific, Massachusetts, USA) combined with 5% foetal bovine serum (ThermoFisher scientific), 1mM ethylenediaminetetraacetid acid (EDTA) (Sigma Aldrich, Saint Louis, USA) and 25mM 4-(2-hydroxyethyl)-1-piperazineethanesulfonic acid (HEPES) (ThermoFisher scientific) at 37 degrees Celsius. These samples were stirred four times, 20 minutes at a time separating each suspension. In order to minimise eventual LPL contamination of the IEL suspension we discarded the fourth cell suspension. A 100 μm and a 30 μm nylon mesh strainer (BD Biosciences, New Jersey, US) were used to filter the first 3 IEL cell suspensions. After IEL isolation, in order to isolate LPL, the biopsies were processed with 100 U/mL collagenase type VIII (Sigma Aldrich, C-2139) and DNAse I (final concentration: 0,1mg/mL) (Sigma Aldrich, D-5025) while stirred for 90 minutes at 37 degrees. Again the same 100 μm and a 30 μm nylon mesh strainer were used to filter the LPL suspension. Simultaneously the whole blood samples were also lysed with 0.8% ammonium chloride, NH_4_Cl, (Sigma-Aldrich) and filtered through the same type of filters. Originally when the study was conceived and executed we planned on separating the epithelial and lamina propria cells by using previous described methods (3) but at the time of analyses a review of a study from Spain demonstrated the difficulties in LPL and IEL separation with possible contamination of the latter from the former (33) which is why the LPL and IEL were merged together.

### Flow cytometry

Lymphocyte populations were analysed by 12-color flow cytometry. The fluorochrome conjugated monoclonal antibodies used are presented in **Supplementary Table 1**. Sample acquisition was performed on BD FACSAria™III III (BD Biosciences, Franklin Lakes, New Jersey, US) flow cytometer. Subsequent data analysis was performed with Kaluza software version 2.1 (Beckman Coulter, Brea, USA). Unstained samples and ``fluorescence minus one`` controls were used as internal gating controls. Major lymphocyte population percentages refer to proportions of the lineage marker excerpt CD45^+^ from the side scatter (SSC), forward scatter (FSC). Cell gating and data collection were performed in a blinded manner.

### Gating strategy

Major lymphocyte populations were identified as follow; T cells (CD45^+^CD3^+^), B cells (CD45^+^CD19^+^), NK cells (CD3^-^CD56^bright^CD16^-^ or CD3^-^CD56^dim^CD16^+^). T helper cells were identified as CD3^+^CD4^+^CD8^-^, T cytotoxic cells as CD3^+^CD4^-^CD8^+^, and MAIT cells as CD3^+^TCRVa7.2^+^CD161^+^. The marker CD45R0 was used as a memory marker and CD69 and CD154 as markers of activation. The CD161 marker was also used in order to differentiate a pro-inflammatory subset of NK cells. The gating strategies are presented in **Supplementary Presentation 2**.

### Immunohistochemistry

Formalin-fixed tissues embedded in paraffin were mounted and de-paraffinized according to standard laboratory procedure. The number of individuals in whom paraffin tissues were available; au-CC: 20, rb-CC: 18, HC: 16, ref-CC: 14. Consecutive slides were stained with anti-CD3 (clone LN10, 1/200 dilution) (Leica Biosystems, Wetzlar, Germany) while haematoxylin was used as a counterstain. The Nikon E800 microscope (Nikon instruments Inc. Tokyo, Japan), equipped with a x 40 objective lens, and connected to the software NIS elements (Nikon instruments Inc. Tokyo, Japan) was used to acquire digital photos. The ImageJ programme (https://imagej.nih.gov/) was used in order to manually quantify the numbers of CD3^+^ lymphocytes in a minimum of eight unique fields of vision (**Supplementary Image 1**) in three consecutive slides. After un-blinding the data a median value of CD3^+^ cells was calculated for each individual. By using the flow cytometry percentages and the median value of CD3^+^ IHC cells we were then able to calculate the absolute number of CD3^+^ cells in each individual and the median values for each group (au-CC, HC, ref-CC, rb-CC) (34).

### Statistics

The nonparametric Mann-Whitney *U* test was used for non-paired analysis between groups and the Wilcoxon test was used for pairwise comparisons within groups. The GraphPad Prism (San Diego, USA) was used in order to plot the data. The Statistica version 12.7 (Palo Alto, USA) was used for statistical analysis. A two-sided level of P<0.01 was used for statistical significance and a two-sided level of 0.01<P<0.05 was considered a trend.

## Results

### Decreased circulating MAIT cells, double negative T cells and NK cells in active untreated collagenous colitis

**Figure 1:**
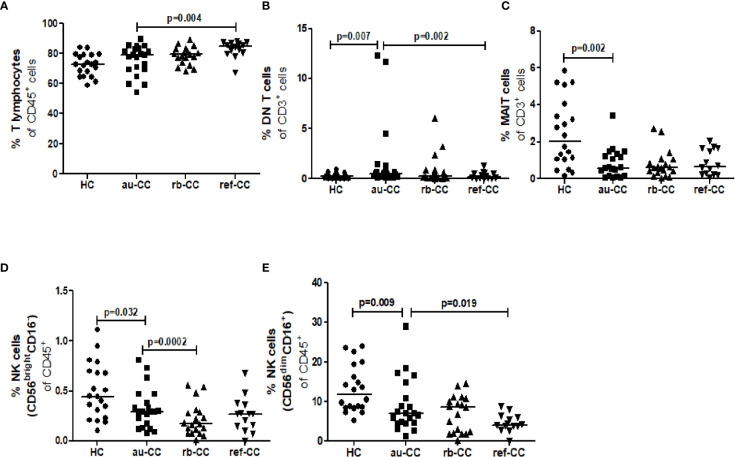
Cell population percentages of T lymphocytes **(A)**, DN T cells **(B)**, MAIT cells **(C)**, CD56brightCD16- NK cells **(D)** and CD56dimCD16+ NK cells **(E)** in the blood of CC patients. Analysis performed with flow cytometry. Statistically significant differences are p<0.01, statistical trends are p<0.05. au, active/untreated; CC, collagenous colitis; HC, healthy controls; rb, remission/budesonide.

**Figure 2:**
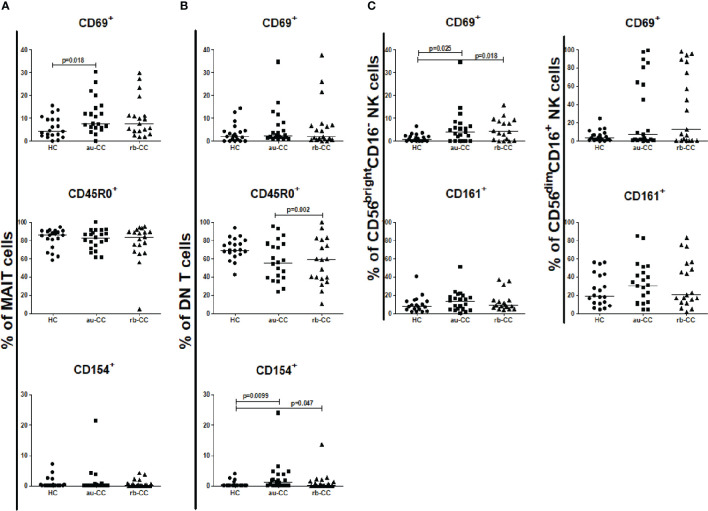
Frequencies of peripheral activated and memory subpopulations of MAIT cells **(A)**, DN T cells **(B)**, CD56brightCD16- and CD56dimCD16+ NK cells **(C)** in collagenous colitis. Analysis performed with flow cytometry. Statistically significant differences are p<0.01, statistical trends are p<0.05. au, active/untreated; CC, collagenous colitis; HC, healthy controls; rb, remission/budesonide.CD69, CD154 and CD161 are activation markers and CD45R0 memory marker.

The percentages of circulating MAIT cells were lower in au-CC patients than in HC (**Figure 1C**). The percentages of T lymphocytes did not differ between au-CC and HC **(Figure 1A)**. The majority of circulating MAIT cells expressed CD45R0, *i.e.* a memory phenotype, in all groups (**Figure 2A**). There was a trend (p=0.018) of increased percentages of CD69 expressing MAIT cells in au-CC patients compared to HC (**Figure 2A**), while the CD154+ MAIT cell frequencies were similar in au-CC and HC (**Figure 2A**). The CD4^-^CD8^+^ subset of MAIT cells were most abundant, followed by DN MAIT and CD4^+^CD8^-^ MAIT cells in all groups (data not shown). In summary, active untreated collagenous colitis is characterised by decreased percentages of circulating MAIT cells.

There was an expansion of circulating DN T cells in au-CC patients compared with HC (**Figure 1B**) and an increase in the DN T cell subset expressing CD154 in au-CC compared to HC (**Figure 2B**). The levels of activation, *i.e.* CD69 expression, and the percentages of DN T cells with a memory phenotype, *i.e.* CD45R0^+^, were similar in the peripheral blood of au-CC and HC (**Figure 2B**).

Within the circulating NK cell population there were lower percentages of CD56^dim^CD16^+^ NK cells and a trend (p=0.032) of lower percentages of CD56^bright^CD16^-^ NK cells in au-CC compared to HC (**Figures 1D, E**). There was a trend (p=0.025) towards increased percentages of CD56^bright^CD16^-^ NK expressing the activation marker CD69 in au-CC compared to HC (**Figure 2C**). The expression of CD161 did not differ between any of the NK cell subsets. In conclusion, reduced percentages of NK cells in the circulating lymphocyte compartment were seen in active untreated CC and the reduction of the CD56^dim^CD16^+^ NK subset was most pronounced.

**Figure 3:**
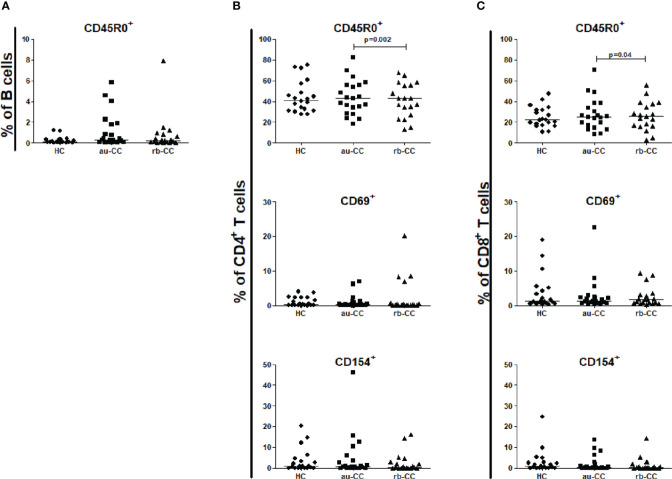
Frequencies of peripheral activated and memory subpopulations of B lymphocytes **(A)**, CD4+ T helper cells **(B)** and CD8+ cytotoxic T cells **(C)** in collagenous colitis. Analysis performed with flow cytometry. Statistically significant differences are p<0.01, statistical trends are p<0.05. au, active/untreated; CC, collagenous colitis; HC, healthy controls; rb, remission/budesonide. CD69 and CD154 are activation markers and CD45R0 memory marker.

No differences were found between CD19^+^ B cells, CD4^+^ T cells or CD8^+^ T cells in peripheral blood when comparing au-CC and HC (**Supplementary Table 2**), neither regarding frequencies nor their activation and memory status (**Figure 3**).

In rb-CC compared to au-CC the frequencies of memory, CD45R0^+^, CD4^+^ and CD8^+^ T cells were decreased (**Figure 3**).

### Increased numbers of mucosal T cells in active untreated collagenous colitis

**Figure 4:**
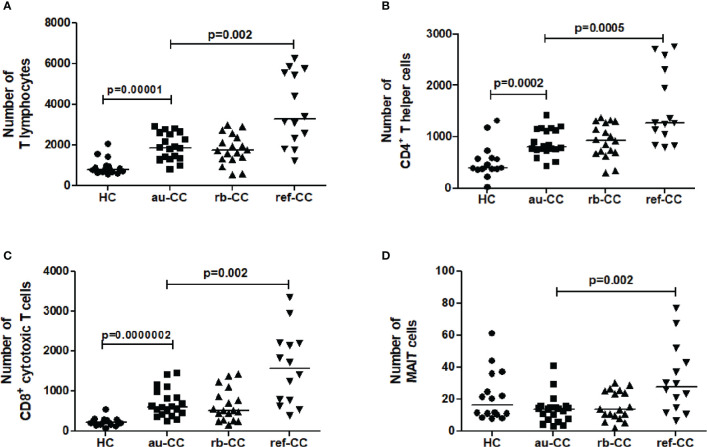
Numbers of lymphocyte subpopulations of T lymphocytes **(A)**, CD4+ T helper cells **(B)**, CD8+ cytotoxic T cells **(C)** and MAIT cells **(D)** in the colonic mucosa of CC patients. Analysis performed with a combination of immunohistochemistry and flow cytometry. HC, healthy controls; CC, collagenous colitis; au, active untreated; rb, remission budesonide; ref,refractory Statistically significant differences are p<0.01, statistical trends are p<0.05.

There were increased numbers of T cells in the mucosa of au-CC compared to HC (**Figure 4A**). Both CD4^+^ T helper cells and CD8^+^ cytotoxic T cell numbers were increased in au-CC compared to healthy controls (**Figures 4B, C**), which also were reflected in higher percentages of these subsets (**Supplementary Table 3**). The percentages of mucosal B cells were not affected in au-CC (**Supplementary Table 3**). NK cells constituted a minor fraction of mucosal lymphocytes in CC and HC (**Supplementary Table 3**). The total number of mucosal MAIT cells did not differ between au-CC and healthy controls (**Figure 4D**). However, there was a trend towards decreased numbers of CD4^+^CD8^-^ MAIT cells (p=0.019) and CD4^-^CD8^+^ MAIT cells (p=0.010) in au-CC patients compared to HC (data not shown).

**Figure 5:**
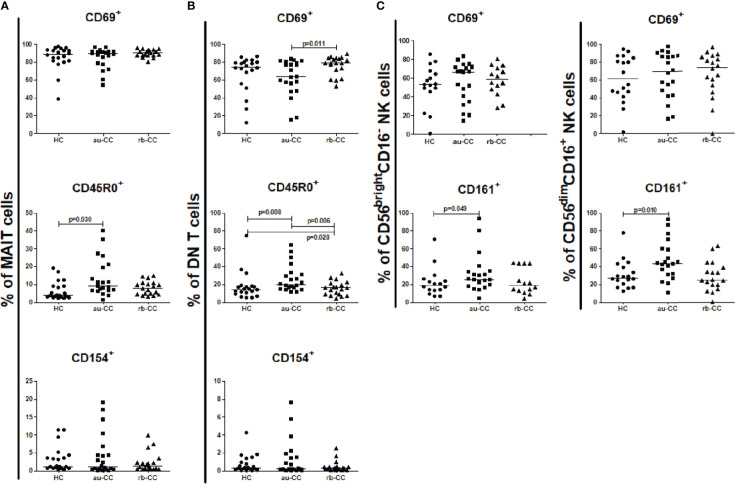
Frequencies of mucosal activated and memory subpopulations of MAIT cells **(A)**, DN T cells **(B)**, CD56brightCD16- and CD56dimCD16+ NK cells **(C)** in collagenous colitis. Analysis performed with flow cytometry. Statistically significant differences are p<0.01, statistical trends are p<0.05. au, active/untreated; CC, collagenous colitis; HC, healthy controls; rb, remission/budesonide. CD69, CD154 and CD161 are activation markers and CD45R0 memory marker.

We found no differences in the percentage of the expression of the activation marker CD154 or tissue resident CD69^+^ in mucosal MAIT cells in au-CC compared to HC, but there was a trend (p=0.030) towards increased percentages of memory MAIT cells (CD45R0^+^) in the mucosa of au-CC (**Figure 5A**). The same pattern with unaltered expression of CD69 and CD154 and expansion of CD45R0^+^ memory cells in au-CC was observed with in the DN T cell mucosal population. The CD45R0^+^ DN T cell populations were contracted during budesonide but did not return to levels observed in HC (**Figure 5B**).

**Figure 6:**
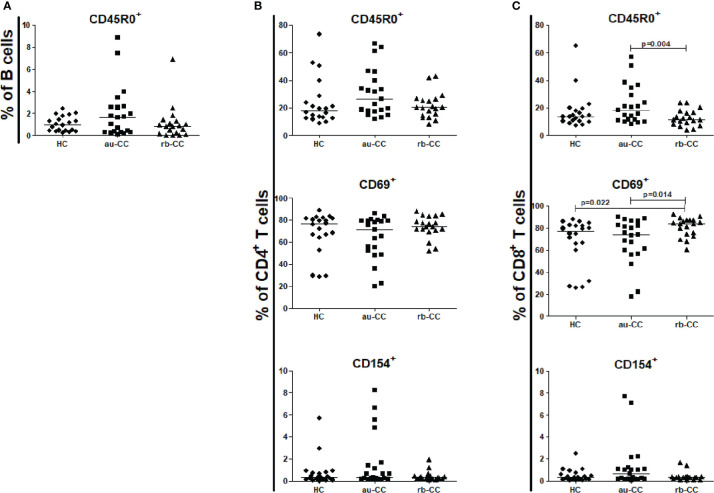
Frequencies of mucosal activated and memory subpopulations of B lymphocytes **(A)**, CD4+ T helper cells **(B)** and CD8+ cytotoxic T cells **(C)** in collagenous colitis. Analysis performed with flow cytometry. Statistically significant differences are p<0.01, statistical trends are p<0.05. au, active/untreated; CC, collagenous colitis; HC, healthy controls; rb, remission/budesonide. CD69 and CD154 are activation markers and CD45R0 memory marker.

CD4^+^ T cells, CD8^+^ T cells and CD45R0^+^ memory B cells did not differ between au-CC and HC (**Figure 6**). CD4^+^ T helper cells and CD8^+^ T cytotoxic cells were not more activated with the CD154 activation marker (**Figures 6B, C**) in au-CC compared to HC. The frequencies of CD69 expressing tissue resident CD4^+^ T cells were similar in au-CC and HC (**Figure 6B**). Within the CD8^+^ cytotoxic T cell population the fraction CD69^+^ tissue resident cells tended to be increased in rb-CC when compared to HC (p=0.02) (**Figure 6C**). When comparing au-CC and rb-CC patients the only differences were between CD8^+^ T cytotoxic cells that showed a decrease in memory CD45R0^+^ cells (p=0.004) and a trend towards increased CD69 tissue resident marker in rb-CC (p=0.014) (**Figure 6C**).

### Low frequencies of mucosal NK cells in active untreated collagenous colitis

The percentage of mucosal NK cells were low and the level of activation did not differ between au-CC and HC. While we did not find any differences in the percentages of CD56^bright^ CD16^-^ and CD56^dim^ CD16^+^ (**Supplementary Table 3**), we did find a trend towards a significant increase of CD56^bright^CD16^-^ NK cells expressing CD161 in au-CC mucosa compared to HC (p=0.049), as well as a trend for CD161^+^ cells among CD56^dim^CD16^+^ NK cells (p=0.010) (**Figure 5C**).

### Budesonide refractory collagenous colitis is characterized by persistent high numbers of mucosal T and MAIT cells

Irrespectively of being on or off budesonide, refractory CC patients showed increased numbers of mucosal CD3^+^ T cells compared to au-CC (**Figure 4**). Within the T cell compartment, CD8^+^ cytotoxic T cells and CD4+ T helper cells were both increased in ref-CC compared to patients with au-CC (**Figure 4**). There was also an increase in MAIT cells in refractory CC compared to au-CC. Among MAIT cells, most cells were CD4^-^CD8^+^ (data not shown). The percentages of cells expressing activation or memory markers were similar when comparing refractory to au-CC patients (data not shown). In summary, the budesonide refractory mucosa differs from the au-CC mucosa by higher numbers of infiltrating CD4^+^ T cells, CD8^+^ T cells and MAIT cells.

## Discussion

In this prospective study, mucosal and blood lymphocytes were phenotyped by multi-colour flow cytometry in collagenous colitis individuals with active untreated disease and during budesonide induced remission and compared to healthy controls. The absolute numbers of mucosal CD3^+^ lymphocytes were calculated by a combination of histological and flow cytometry data. In untreated active collagenous colitis, the lymphocyte composition of the colonic mucosa showed increased T cells, with an increase of both CD8^+^ cytotoxic and CD4^+^ helper T cells. These changes persisted during budesonide-induced remission. Absolute numbers of MAIT cells did not differ in the mucosa of untreated active collagenous colitis patients compared to healthy controls. In the mucosa of budesonide refractory collagenous colitis, we found higher numbers of CD8^+^ and CD4^+^ T cells and MAIT cells when compared to untreated active collagenous colitis patients.

MAIT cells are a type of invariant T cell population, characterised as CD3^+^TCRVa7.2^+^CD161^+^, which are present in the blood and in the gastrointestinal mucosa of humans (22). They are mainly activated by microbial vitamin B metabolites (35), *via* the major histocompatibility complex related molecule MR1 and considered cells of the innate immune system (36). We found decreased percentages of circulating MAIT cells in individuals with active untreated collagenous colitis when compared to controls. Decreased percentages of MAIT cells in blood have previously been described in patients with classic inflammatory bowel disease (25, 26, 37–39), celiac disease (38) and in *Helicobacter pylori* infection (40). Among circulating MAIT cells, we observed an expansion of cells expressing the early activation marker CD69 in untreated active collagenous colitis patients, a finding similar to what has been described in classical inflammatory bowel disease (25, 37), and may be an indication that they have an active role in disease pathogenesis. Reduction of circulating MAIT cells has been considered either secondary to migration of MAIT cells to the inflamed mucosa (25) or due to increased apoptosis (37).

In the colonic mucosa we discovered higher absolute numbers of MAIT cells in refractory patients compared to untreated active collagenous colitis patients. Also in classical inflammatory bowel disease patients tend to have increased percentages of mucosal MAIT cells compared to controls (25, 26, 41) but studies on MAIT cells in cortisone induced remission in IBD are missing. There is a report of chronic obstructive pulmonary disease patients, where MAIT cells were reduced in isolates from bronchial biopsies and less activated in patients receiving local corticosteroids compared to patients not receiving inhalation steroid therapy (42). It is possible that MAIT cells might be involved in the mechanisms over-riding the effect of budesonide in refractory collagenous colitis. Upon contact with microbes, MAIT cells can act both as cytotoxic-and pro-inflammatory cytokine producing cells (43) giving them a role against pathogen invasion of the mucosa. Since a luminal substance is believed to cause collagenous colitis; the increase of MAIT cells in refractory CC might hint towards a microbial culprit. In the mucosa we have found that most MAIT cells are CD4^-^CD8^+^ with the double negative and CD4^+^CD8^-^ populations being much less prevalent which is a common finding in MAIT cells and mirrors their effector properties (44). At the same time studies have shown that not all CD4^+^CD8^-^TCRVa7.2^+^CD161^+^ mucosal cells stain for MRI tetramers indicating that not all are genuine MAIT cells (45, 46) making this population even less common in this compartment.

NK cells, previously considered a homogenous population, are nowadays divided into two main functional categories; CD56^bright^CD16^-^ and CD56^dim^CD16^+^ (47). The CD56^bright^CD16^-^ subset consists of immune-modulatory, cytokine producing cells (48) and has been described to be important in maintaining intestinal homeostasis (49). The CD56^dim^CD16^+^ subset is the most abundant in the peripheral circulation (50), and has cytotoxic capacity (48). We observed changes in circulating NK-cells in untreated active collagenous colitis patients, with reduced percentages of both CD56^dim^CD16^+^ and CD56^bright^CD16^-^ NK cells compared to HC. Reduced percentages of circulating NK cells (CD56^+^CD16^+^) have also been described in severe Crohns disease (51). The reduction in circulating NK cells was in this case suggested to be secondary to increased migration into the mucosa (21). However, we found no differences in mucosal NK-cell percentages in untreated active or during budesonide remission collagenous colitis compared to healthy controls. Others have instead found increased lamina propria NK cells (CD56^+^CD16^+^) in the mucosa of inflammatory bowel disease patients, that are restored to normal levels during azathioprine treatment (21). Based on our findings, it is unlikely that NK cells contribute significantly in the pathogenesis of collagenous colitis, since mucosal percentages of NK-cell subsets during CC active disease were unaffected and the absolute numbers of NK cells were very low. We did notice a decrease of CD161^+^ expression in CD56^bright^CD16^-^ cells in refractory collagenous colitis mucosa. Such a decrease in CD161^+^ NK cells has been previously found in patients with early HIV infection (52) as well as in patients with metastatic melanoma patients (53). Since these CD161^+^ cells are considered pro-inflammatory (54), this decrease might indicate a different innate response in refractory CC patients.

Our finding of increased numbers of mucosal CD4^+^ T helper cells in untreated active collagenous colitis patients is in line with a previous study (11). In another study, that included a mixed cohort of treated/untreated collagenous colitis patients, the lamina propria CD4^+^ percentages were equal to healthy controls but showed signs of increased activation in collagenous colitis (3). In that case the difference could be due to a less defined population of collagenous colitis patients compared to ours, where all patients were originally active and untreated. We did not observe any increased activation in CD4^+^ T cells when using CD154 marker. CD4^+^ T helper cells are the most abundant lymphocytes in human mucosa and have a pivotal role in recruiting and coordinating immune responses (31). In classical IBD CD4^+^ T cells in the presence of commensal gut bacteria have been considered pathogenic (55). We confirm previous findings that peripheral CD4^+^ T cells do not differ in active collagenous colitis or during remission (11). No signs of increased activation of circulating lymphocytes were found and were not expected, since collagenous colitis is not characterized by systemic inflammation.

The increase of CD8^+^ cytotoxic T cells in the mucosa of collagenous colitis patients with active disease has also been thoroughly described (3, 11, 56). The cell cycle marker Ki67 has been previously used to identify increased activated cycling CD8^+^ T cells (3). We found no differences in the CD8^+^ T cell expression of CD154 activation marker in untreated active collagenous colitis patients compared to healthy controls. We found an abundant expression of CD69 in mucosal T cells in our study which is explained by that CD69 is mainly expressed by tissue resident memory T cells (31) and not only activated mucosal cells. The increased CD8^+^ T cell CD69 expression in rb-CC individuals may be associated with reduced recruitment of CD8^+^ T cells from the circulation after budesonide induced remission (57). It has been postulated that autoreactive CD8^+^ cytotoxic T cells initiate the inflammatory process in classical inflammatory bowel diseases (58). In our study, increased CD8^+^ T cells persisted during remission with budesonide. However, the proportion of CD45R0^+^ effector memory cells decreased, and it can be hypothesized that the shift within the CD8^+^ mucosal T cell population towards a larger fraction of CD69^+^, potentially tissue resident memory T cells, are important to counteract relapse. This is due to the ability of tissue resident memory cells to locally control infections and coordinate the responses of the innate and adaptive immune system (59). Finally only healthy controls had a CD4^+^/CD8^+^ quotient with a median ratio of 2.5, which is considered normal for the colon (60) while the rest of the CC groups had median ratios ranging between 1.2 and 1.7. This might indicate the pathological dominance of CD8^+^ cytotoxic T cells in the collagenous colitis colonic mucosa irrespectively of clinical status.

We observed an expansion of the percentages of circulating and mucosal DN T cells in untreated active collagenous colitis patients, a finding in agreement with a previous study (5). In general, the vast majority of the DN T cells in humans express the gamma/delta T cell receptor instead of the alpha/beta expressed by conventional CD4^+^ T helper and CD8^+^ cytotoxic T cells. The gamma/delta marker was not included in our flow cytometry panel. However, it is possible that the expanded population of DN T cells represents gamma/delta invariant T cells that have a role in mucosal barrier protection (61) and could be of interest in future studies of collagenous colitis.

Budesonide therapy is the golden standard of collagenous colitis treatment with approximately 80% of patients achieving remission (8, 9). At the same time refractory patients, even though they represent a small fraction of all CC patients (10), pose a significant problem for both the patient and the treating doctor. This is the first time to our knowledge that this number of budesonide refractory patients were studied on a cellular level and compared to patients responsive to budesonide treatment. We found that most of the cell populations studied were increased in refractory patients and different T cell subsets (CD4^+^, CD8^+^ and MAIT) constituted the vast majority of mucosal lymphocytes in refractory collagenous colitis. Our findings suggest that adaptive immune mechanisms contribute to the failure of budesonide in collagenous colitis. At the same time MAIT cells are more readily categorised as innate T cells and other types of innate cells such as eosinophilis (11) and mast cells (14) have been associated with collagenous colitis. Furthermore, innate lymphoid cells (ILC) type 1 and type 2 have recently been suggested to play a role in intestinal inflammation due to their increased presence in mucosal biopsies of inflammatory bowel disease patients (62). ILC encompass a large family of cells that include NK cells and share similarities with T cells but they do not express the antigen specific T cell receptor (63). A better understanding of the role of MAIT cells and other innate lymphocyte subsets in collagenous colitis could shed new light on disease mechanisms in collagenous colitis and lead to new treatments. Concerning treatment of budesonide- refractory cases, different studies on methotrexate (64–66) and azathioprine (67, 68) have yielded mixed results. The utilization of anti-TNF agents have been more consistent with approximately 70% achieving remission or response (67, 69, 70). The increase of predominantly T cells in refractory collagenous colitis patients suggests that T cell targeting treatment could be efficient. While small case studies with vedolizumab (71, 72) have shown promising results, there is still no mainstay in refractory CC treatment and randomized controlled prospective studies on a multicentre level need to be made especially considering the findings of our study.

This is the first study to delineate circulating and mucosal MAIT and NK cells in collagenous colitis patients and add to the complexity of the disease pathophysiology. One of the strengths of our study is the prospective design, investigating the same individuals during active disease and after budesonide induced remission, thus reducing the problem of biological diversity. Also, the comparison of patients with active untreated disease with budesonide refractory patients has never been done before in this magnitude. Although our cohort of patients can be considered large relative to the low prevalence of collagenous colitis, a larger number of patients would have improved the statistical power of the study. In this study we presented both the percentages of different cell populations analysed by flow cytometry and the absolute number of mucosal lymphocytes by calculation by using a combination of immunohistochemistry and flow cytometry. A loss of a large proportion of CD3^+^ cells has been described during flow cytometry compared to IHC but the cells recovered are considered to be representative (34). We believe that this quantification adds an important aspect to our research. During the period of sample collection in such a longitudinal study, which took our team 3 years to complete, the knowledge is increasing. New markers characterizing MAIT cells have been discovered but at the same time it is, to our knowledge, this is the first time CD161 and Va7.2 have been analyzed in collagenous colitis and it is possible to compare our results with other studies of different IBD where tetramers not have been used. Further limitation of the study is that our control group was not age matched due to the clinical practice in Sweden to include colorectal cancer screening patients at the age of 60 years. All the patients were of Caucasian ethnicity, which might mean that the results are not representative for other ethnicities.

## Conclusion

Collagenous colitis is a chronic inflammatory bowel disease with a complex underlying pathology. While previous studies have shown that there are immunological differences between healthy individuals and collagenous colitis patients, our study expands this knowledge with the analysis of MAIT and NK cells. We find lower percentages of circulating MAIT and NK cells in untreated active collagenous colitis patients, while neither the mucosal percentage of NK cells nor the percentage and number of MAIT cells differ compared to HC. The pronounced differences between MAIT cells, CD4^+^ and CD8^+^ T cells between active collagenous colitis patients that respond to budesonide and budesonide non-responders indicate that separate immunological mechanisms could be present that might require different types of immunomodulating treatments.

## Data availability statement

The raw data supporting the conclusions of this article will be made available by the authors, without undue reservation.

## Ethics statement

Informed written consent was obtained from all patients and healthy controls. Ethical approval was issued by the regional ethical committee at Linköping University Hospital with registry number Dnr 2012/216-31. The patients/participants provided their written informed consent to participate in this study.

## Author contributions

ND, MS and AM designed the study. ND performed the experiments. ND and AM enrolled and sampled he patients and volunteers who participated in the study. SI contributed to patient diagnosis and pathological analysis. ND and MS analysed the data. ND, SN, and MS wrote the manuscript. ND prepared the figures and all supplementary material. All authors reviewed and approved the final version of the manuscript.
