# Supplementary figures and images for: Mucosa associated invariant T and natural killer cells in active and budesonide treated collagenous colitis patients
Source: Front Immunol. 2022 Dec 15;13:981740. doi: 10.3389/fimmu.2022.981740 (PMC9798420; doi:10.3389/fimmu.2022.981740)

**SI Figure 1**

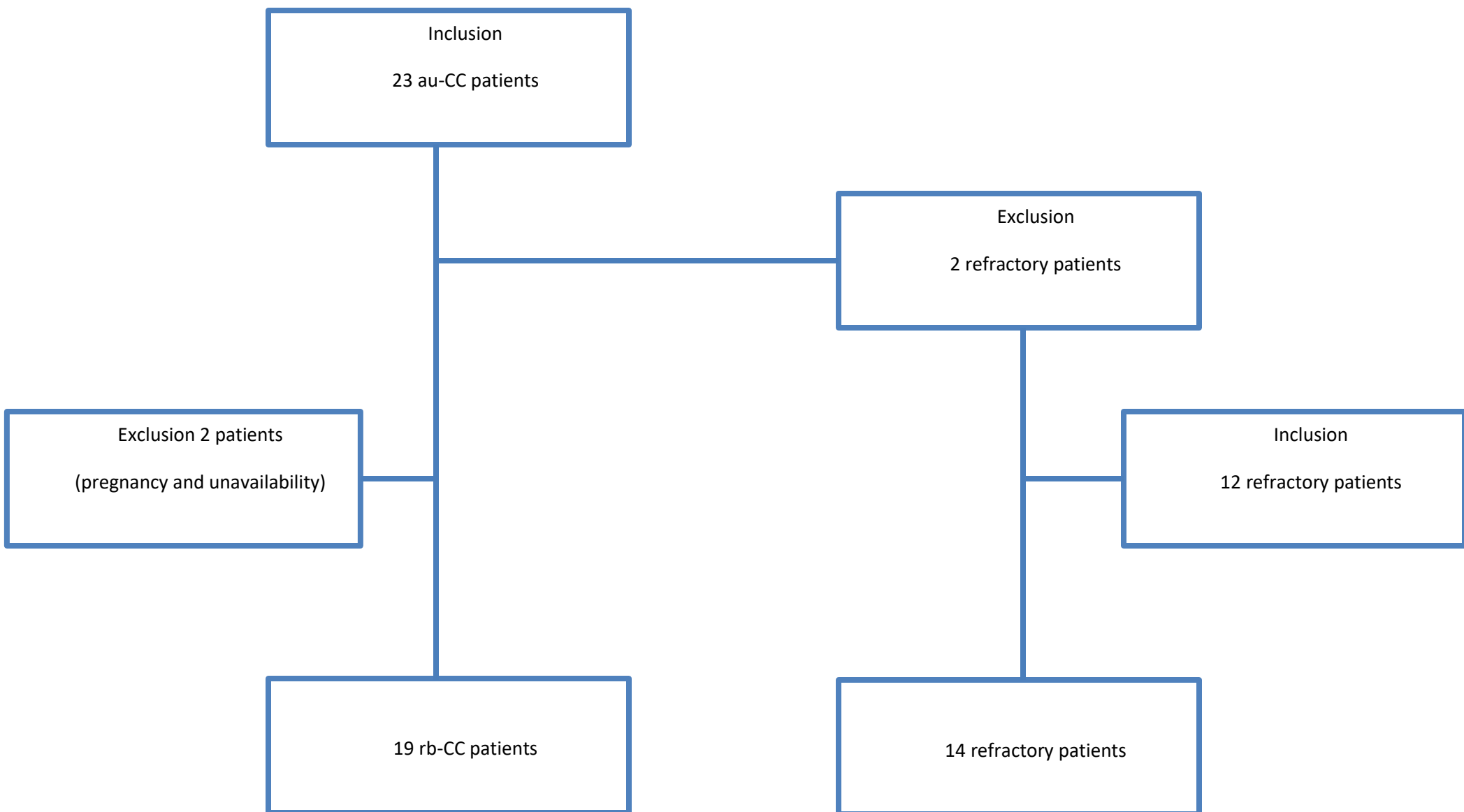

Supplement: Supplementary file 1 [file Presentation_1.pdf]

SI Fig. 2:

A

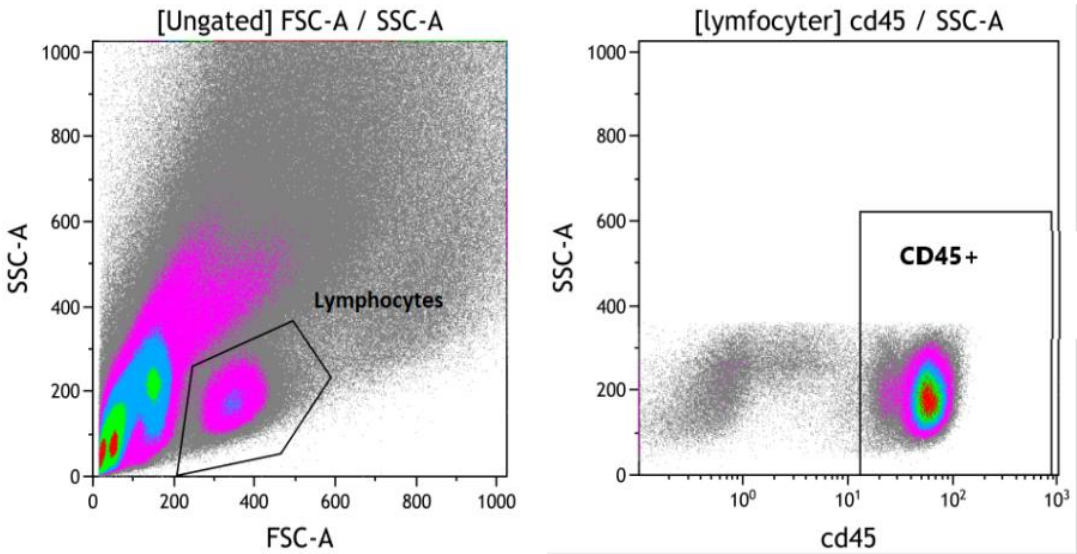

B

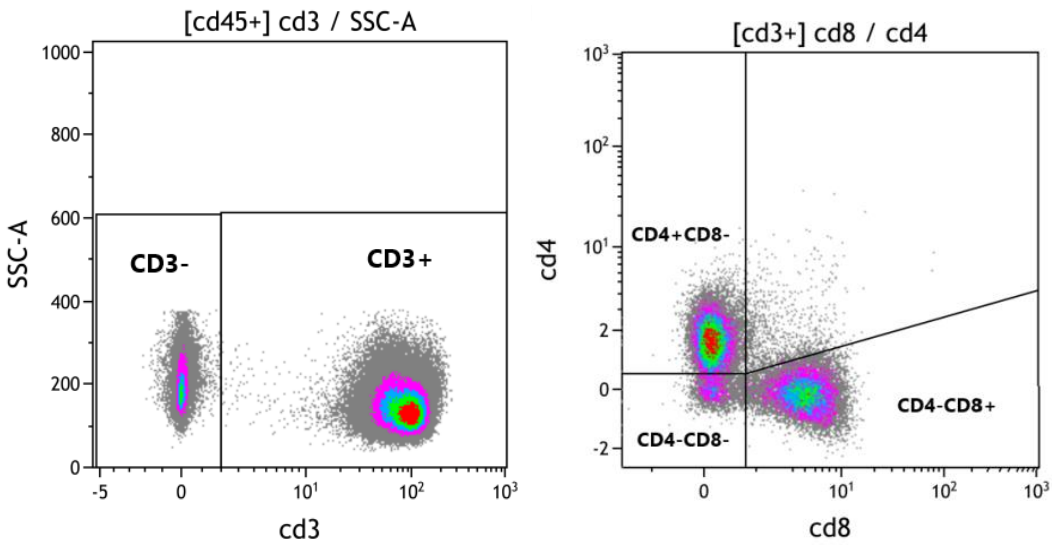

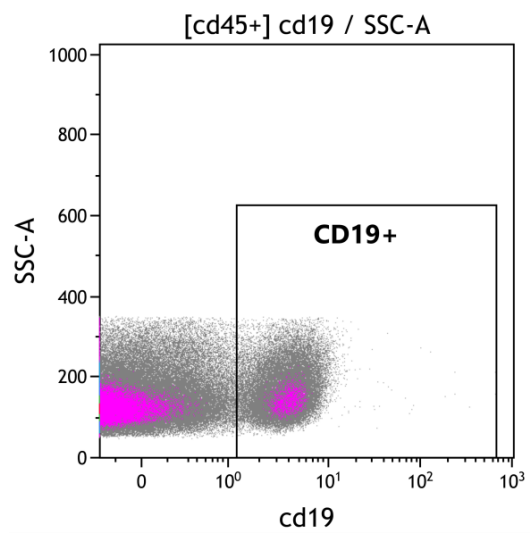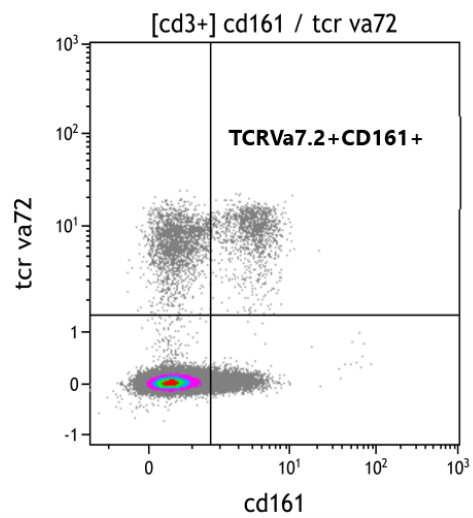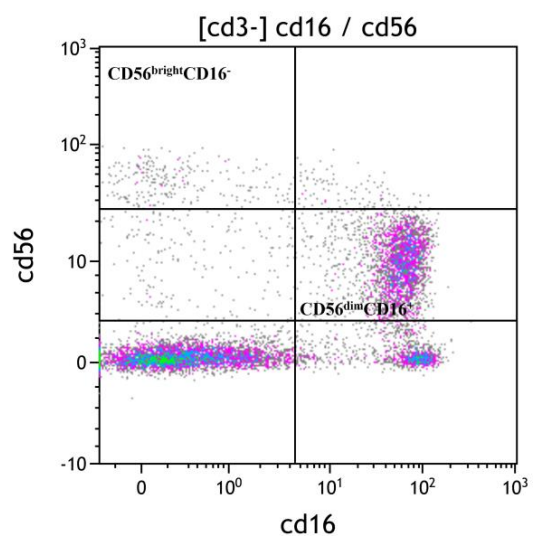

C

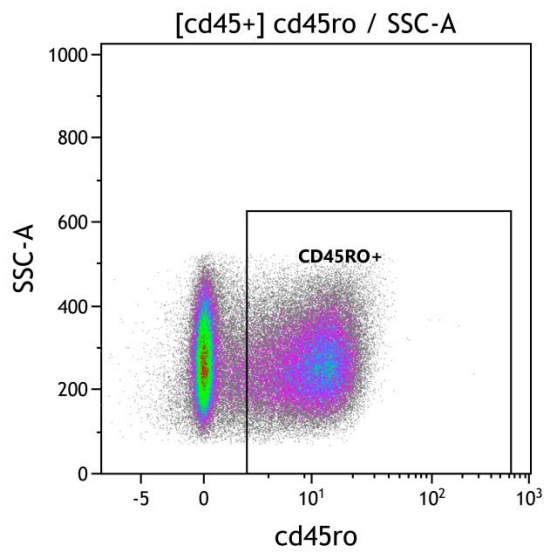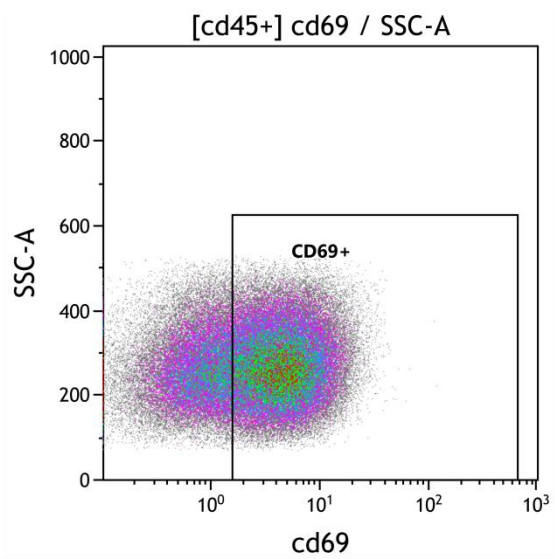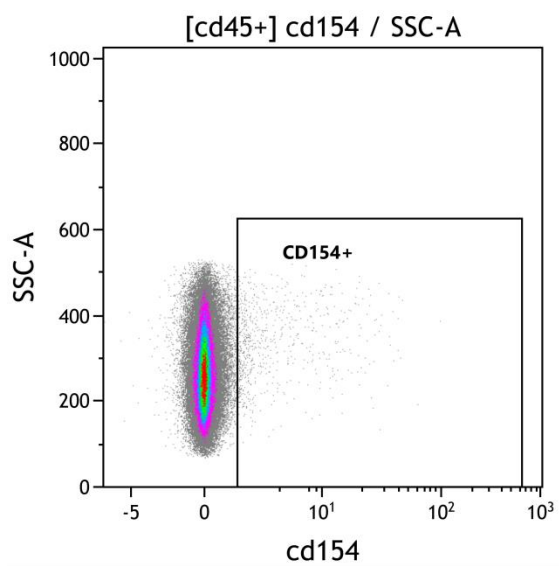

Supplement: Supplementary file 2 [file Presentation_2.pdf]

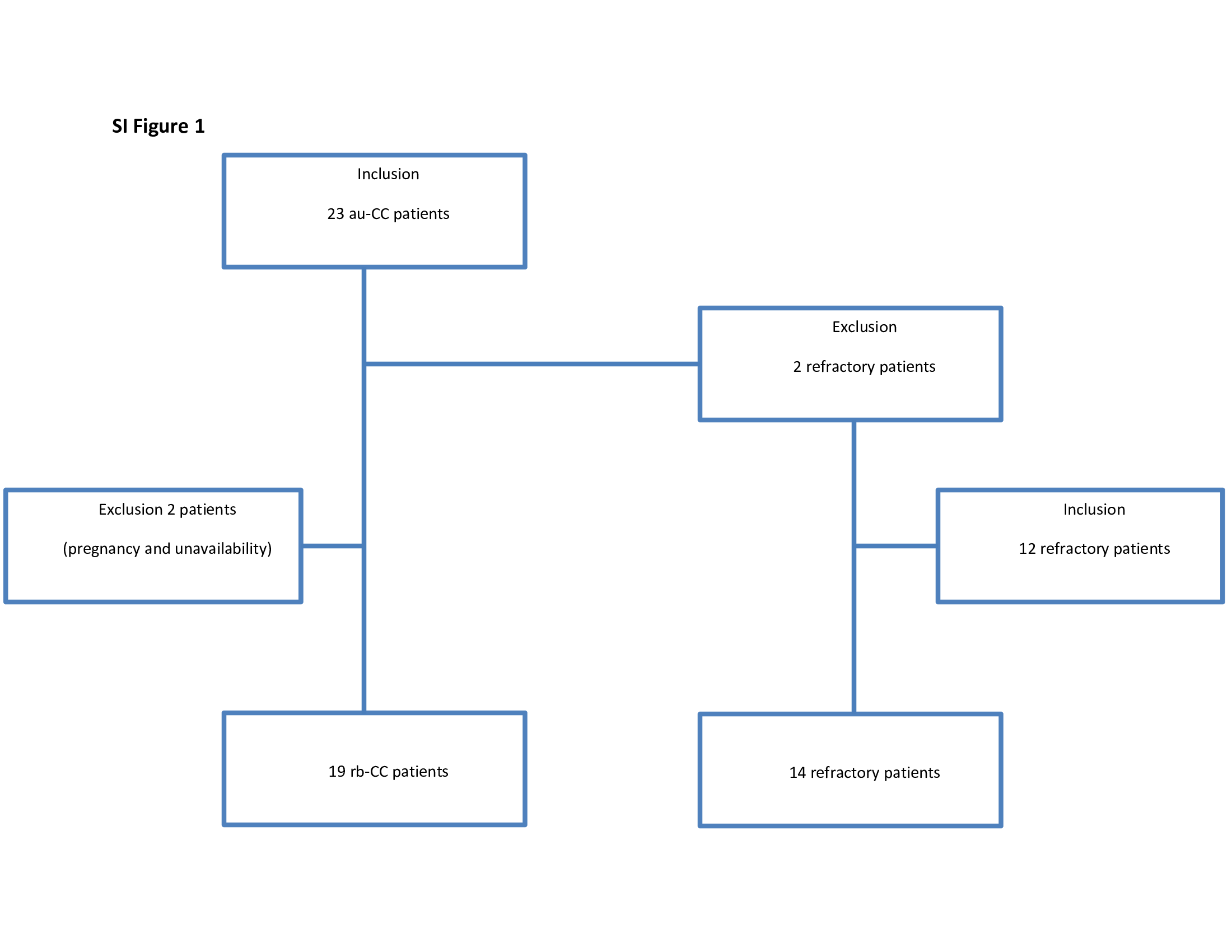

Supplement: Supplementary file 3 [file Image_1.tiff]
