# Supplementary material for: Mucosa associated invariant T and natural killer cells in active and budesonide treated collagenous colitis patients
Source: Front Immunol. 2022 Dec 15;13:981740. doi: 10.3389/fimmu.2022.981740 (PMC9798420; doi:10.3389/fimmu.2022.981740)
Supplement: Supplementary file 4 [file Table_1.docx]

| Antibodies | Fluorophore | Clone | Company |  |
| --- | --- | --- | --- | --- |
| Anti-CD45 | FITC | HI30 | Biolegend | San Diego, CA |
| Anti-CD45R0 | PerCP-Cy5.5 | UCHL1 | Biolegend | San Diego, CA |
| Anti-CD154 | PE | 24-31 | Biolegend | San Diego, CA |
| Anti-CD16 | PE-Cy7 | 3G8 | Biolegend | San Diego, CA |
| Anti-TCR Va7.2 | APC | 3C10 | Biolegend | San Diego, CA |
| Anti-CD3 | PE-CF594 | UCHT1 | BD Bioscience | New Jersey, US |
| Anti-CD4 | APC-H7 | RPA-T4 | BD Bioscience | New Jersey, US |
| Anti-CD8 | AlexaFluor700 | RPA-T8 | BD Bioscience | New Jersey, US |
| Anti-CD69 | BV421 | FN50 | BD Bioscience | New Jersey, US |
| Anti-CD161 | BV510 | DX12 | BD Bioscience | New Jersey, US |
| Anti-CD56 | BV711 | NSAM16.2 | BD Bioscience | New Jersey, US |
| Anti-CD19 | BV786 | SJ25C1 | BD Bioscience | New Jersey, US |

**SI Table 1:**
