# Supplementary material for: Mucosa associated invariant T and natural killer cells in active and budesonide treated collagenous colitis patients
Source: Front Immunol. 2022 Dec 15;13:981740. doi: 10.3389/fimmu.2022.981740 (PMC9798420; doi:10.3389/fimmu.2022.981740)
Supplement: Supplementary file 5 [file Table_2.docx]

**SI Table 2:** Percentages of peripheral lymphocyte subsets in collagenous colitis. Medians and 5^th^-95^th^ percentiles are shown.

| Population | HC | au-CC | rb-CC | ref-CC |
| --- | --- | --- | --- | --- |
| **CD4^+^ T cells**  **(% of CD3^+^**) | 58.6  (33-77.4) | 60.6  (41.5- 83) | 61.3  (34- 86.6) | 58.5  (40.4- 80.1) |
| **CD8^+^ T cells**  **(% of CD3^+^**) | 39.1  (20.8-65.2) | 36.5  (15.7-57.9) | 37.2  (12.6- 65.4) | 40.6  (18.9- 58.7) |
| **CD4^+^/ CD8^+^** | 1.5  (0.6-3.8) | 1.7  (0.7 -5.3) | 1.7  (0.5-6.9) | 1.5  (0.7 -4.1) |
| **CD19^+^ B cells**  **(% of CD45^+^**) | 9  (3.6-15.2) | 8  (0.7-20) | 9.4  (1.4-17.7) | 7  (5.3-16.8) |

HC, healthy controls; CC, collagenous colitis; au, active untreated; rb, remission budesonide; ref, refractory; *** p< 0.00001, ^**^p <0.001,*p<0.01, # p<0.05
