# Supplementary material for: Mucosa associated invariant T and natural killer cells in active and budesonide treated collagenous colitis patients
Source: Front Immunol. 2022 Dec 15;13:981740. doi: 10.3389/fimmu.2022.981740 (PMC9798420; doi:10.3389/fimmu.2022.981740)
Supplement: Supplementary file 6 [file Table_3.docx]

**SI Table 3.** Percentages of mucosal lymphocyte subsets in collagenous colitis. Medians and 5^th^-95^th^ percentiles are shown.

| Population | **HC** | **au-CC** | **rb-CC** | **ref-CC** |
| --- | --- | --- | --- | --- |
| **CD3^+^ T cells**  **(% of CD45^+^)** | 77.3  (55.5-94)  * | 86  (70.7- 91.3)  * | 83.1  (51.4- 91.9) | 81.7  (67- 95) |
| **CD4^+^ T cells**  **(% of CD3^+^)** | 59.6  (15.1-79.23)  * | 50.7  (35.3- 63.7)  * | 54.4  (28.6- 69.9) | 44.7  (18.6- 64.1) |
| **CD8^+^ T cells**  **(% of CD3^+^)** | 23.7  (9.7-35.2)  ** | 35.9  (19.5-50.4)  ** | 31.7  (12.7 52.9) | 38.5  (30- 57) |
| **CD19^+^ B cells**  **(% of CD45^+^)** | 12.7  (0.09-31.9) | 8.9  (4.6- 24.7) | 11.9  (4.6- 43.5) | 11.5  (2.1- 34.6) |
| **NK cells CD56^+^CD16^+^**  **(% of CD45^+^)** | 0.3  (0.04-0.8) | 0.23  (0.01- 0.73) | 0.13  (0- 1.04) | 0.14  (0.02- 0.76) |
| **NK cells**  **CD56^bright^CD16^-^**  **(% of CD45^+^)** | 0.09  (0.02-0.5) | 0.09  (0.02-0.26) | 0.08  (0.007-0.32) | 0.07  (0.07-0.3) |
| **CD56^dim^CD16^+^**  **(% of CD45^+^)** | 0.31  (0.04-0.83) | 0.23  (0.01-0.74) | 0.12  (0-1.04) | 0.14  (0.02-0.76) |

HC, healthy controls; CC, collagenous colitis; au, active untreated; rb, remission budesonide; ref, refractory; ^**^p <0.001,*p<0.01, # p<0.05
